# The effect of optimal load training on punching ability in elite female boxers

**DOI:** 10.3389/fphys.2024.1455506

**Published:** 2024-08-29

**Authors:** Weijia Cui, Yiming Chen, Dexin Wang

**Affiliations:** ^1^ School of Athletic Performance, Shanghai University of Sport, Shanghai, China; ^2^ School of Exercise and Health, Shanghai University of Sport, Shanghai, China

**Keywords:** boxing, punching ability, optimal load training, strength training, training load

## Abstract

Optimal load training is a method of training that aims to maximize power output. This is achieved by arranging optimal loads (optimal ratios of load intensity and load volume) during strength training. The fixed load intensity and number of repetitions employed in traditional strength training. The present study will investigate the applicability of these two load arrangements to female elite boxers. Twenty-four elite female boxers were divided into three groups [optimal load (OL = 8), traditional load (TL = 8) and control group (CG = 8)]. The six-week intervention consisted of strength training with different loading arrangements. The punching ability and strength were tested before and after the intervention. We found that optimal load training enhances a boxer’s punching ability and economy, which aligns with the demands of boxing and is suitable for high-level athletes, whose strength training loads require a more individualised and targeted approach.

## 1 Introduction

In amateur women’s boxing, the significant time structure characteristics and intense confrontation make it a high-intensity sport. Athletes must complete a large number of short, high-explosive movements during the whole match (Three rounds of 3 min each, with a one-min break between rounds). As the rules of boxing have changed and the emphasis on physical fitness has increased in recent years, the style of boxing has shifted from point scoring to intense confrontation. This necessitates that athletes possess highly developed muscular strength and explosive power in order to enhance their punching ability, thereby creating a deterrent to gain an advantage in the game (Piorkowski et al., 2011). Consequently, the current focus of research is on the maximisation of the punching force, punching speed and punching power of boxers, with the objective of improving the punching ability and guaranteeing the effective punching.

Strength is the maximal force a muscle or muscle group can generate against resistance (Marzuca-Nassr et al., 2024). Force is an interaction that changes the motion of an object when unopposed (Olberding and Deban, 2017). Power is the rate at which work is done or energy is transferred (Cormie et al., 2010). Velocity is the rate of change of an object’s position, including direction and magnitude (Horan and Kavanagh, 2012). Speed is how fast an object is moving, measured as distance traveled per unit time (Horan and Kavanagh, 2012). Strength is regarded as the cornerstone of boxers’ confrontation ability, and power output is the embodiment of strength. Athletes’ maximal power output ability is regarded as one of the key factors to win the game (Turner et al., 2011). The successful implementation of techniques and tactics in most sports is based on the athletes’ ability to achieve maximal power output. Furthermore, the power output ability is closely related to the athletic performance. In order to ensure the safety and effectiveness of training, especially for elite athletes, the training load arrangement should be more accurate and targeted based on the consideration of individual differences (Soriano et al., 2015). Therefore, it is important to have a deep understanding of the interrelationship between training variables, including load intensity and load volume, interval time, movement pattern and movement speed, and so forth. A number of studies have demonstrated that the most effective training outcomes are achieved through the utilisation of load arrangements that maximise power output during training, and are therefore defined as optimal training loads (Kawamori and Haff, 2004; Peterson et al., 2004). Training with individual optimal loads represents a strength training method that seeks to optimise the combination of force and speed. This method has been demonstrated to have significant training effects in sports that require explosive movements (Cormie et al., 2011a; Flores et al., 2017). The objective of strength training for boxers is not merely to enhance muscular strength, rather, it is to facilitate the transfer of strength gains from training to improved punching ability (Loturco et al., 2021).

In this study, optimal load training is defined as the optimal ratio of load intensity and load volume that results in the greatest possible power output (Loturco et al., 2017). The monitoring tool used was the GymAware PowerTool system (GYM). Therefore, this study investigated the effects of a 6-week training intervention on the punching ability and strength of elite female boxers by designing a training intervention programme with two different loading regimens (optimal load training and traditional load training) and a control group (performing conventional training).

We hypothesize that following a six-week intervention period, the three subject groups would demonstrate targeted improvements in punching ability and strength. It was further hypothesised that optimal loading training would prove more applicable to boxing and may benefit punching ability, whereas traditional loading training may focus on strength gains.

## 2 Materials and methods

### 2.1 Sample and participants

Twenty-four elite female boxers (age 22.12 ± 2.84 years; height 165.52 ± 4.67 cm; weight 64.56 ± 12.17 kg) were randomly assigned to three groups [optimal load (OL = 8), traditional load (TL = 8) and control group (CG = 8)], specific information is provided in Table 1. Participants reported no history of injury or illness in the 6 months prior to the experiment. They were informed about the procedure and the aim of the study, and subsequently they provided their written consent for participation. Ethical consent was provided by Shanghai University of Sport research ethics committee (approval number: 102772023RT153) and in accordance with the Helsinki declaration.

**TABLE 1:** Basic information sheet for subjects.

	OL	TL	CG
Height (cm)	166.21 ± 3.14	166.13 ± 2.87	167.16 ± 2.74
Weight (kg)	64.67 ± 11.54	62.65 ± 11.94	63.39 ± 14.04
Age (year)	22.4 ± 1.65	22.38 ± 1.58	22.44 ± 1.55
BP (kg)	56.88 ± 5.56	56.88 ± 4.28	56.56 ± 2.48
SQ (kg)	63.13 ± 4.96	61.88 ± 4.28	66.25 ± 4.44

### 2.2 The main experimental steps

The whole experiment was divided into four parts: pre-test, intervention load test, 6-week intervention and post-test. In this study, the athlete’s punching ability was measured by Strike Tec Boxingperformance Tracking (STRIKETEC SENSOR KIT), the GYM is used to test the power output when performing training maneuvers, and the CMJ test used the Smart jump electronic long jump pad for testing.

The optimal training load intensity for the upper and lower limbs of the OL was derived through the intervention load test. This required the subjects to start at a load intensity of 30% 1 RM and complete the test movement three times in a row, with the data recorded through the real-time feedback data from the GYM. This enabled the calculation of the average power, average propulsive power, and peak power of the three movements. Following the completion of the 30% 1 RM load intensity test movement, the subject was required to rest for a period of 5 min before commencing the 40% 1 RM load intensity test. This involved the completion of three consecutive movements, with a further 5 min of rest between each set. The load intensity was increased by 10% 1 RM each time, up to a maximum of 70% 1 RM. During the test, when a significant decrease in average power, average propulsive power, and peak power was observed to be lower than that of the previous set, the test was concluded, and the load that was able to produce the maximum power output was regarded as the optimal load and used as the intervention load (Loturco et al., 2013; Cormie et al., 2011b).

The training intervention comprised a series of exercises, including squats, bench presses, bench pulls and hip thrusts. The load intensity of the OL was the optimal load that could achieve the maximum power output derived from the pre-intervention load test. The load volume was preset to 4-9 repetitions, and the whole process was monitored in real time by the GYM. When the power output decreased significantly, the training of the group ended and entered into a 20 s break between sets. TL are loaded at 61%–66% 1 RM and loaded at 50% of the maximum number of repetitions at that loaded intensity (Sarabia et al., 2017). The OL and TL are completed with six sets of each movement, with a 20-s rest period between sets and a 2–5 min rest period between each training movement (Baker, 2003). The CG underwent routine training.

Outcome measures were: 1) Punching ability: single punching force (SPF), single punching speed (SPS), single punching power (SPP); Average force of continuous punching (CPF), average speed of continuous punching (CPS), average power of continuous punching (CPP); Number of sandbags punch in 10 s (10 PS), Number of sandbags punch in 30 s (30 PS), Number of sandbags punch in 60 s (60 PS) (Smith et al., 2000). 2) Strength: 1 RM bench press (BP), 1 RM squat (SQ), counter movement jump (CMJ).

### 2.3 Statistical analysis

Statistical analyses were conducted with IBM statistics SPSS v26.0 software (SPSS Inc., Chicago, IL, United States). A Shapiro-Wilk test was initially used to assess the normal distribution of the data, which was found to be satisfactory. One-way ANOVA was used to analyze the physical characteristics of the participants across the three groups in terms of age, height, weight BP and SQ. A two-way repeated-measures ANOVA with a Bonferroni *post hoc* test was used to detect significant differences within groups between week 0 and week 6 of training. Differences in improvement effect by group were tested by the one-way repeated-measures analysis of variance with a Bonferroni *post hoc* test. Growth rate = (post-test - pre-test)/pre-test * 100%.

Utilizing G*Power 3.1 software, we took a moderate effect size (*η* ^2^ = 0.059), with a statistical power of 0.8 and a significance level of 0.05. Derived the need for a minimum of 24 subjects.

## 3 Results

**FIGURE 1:**
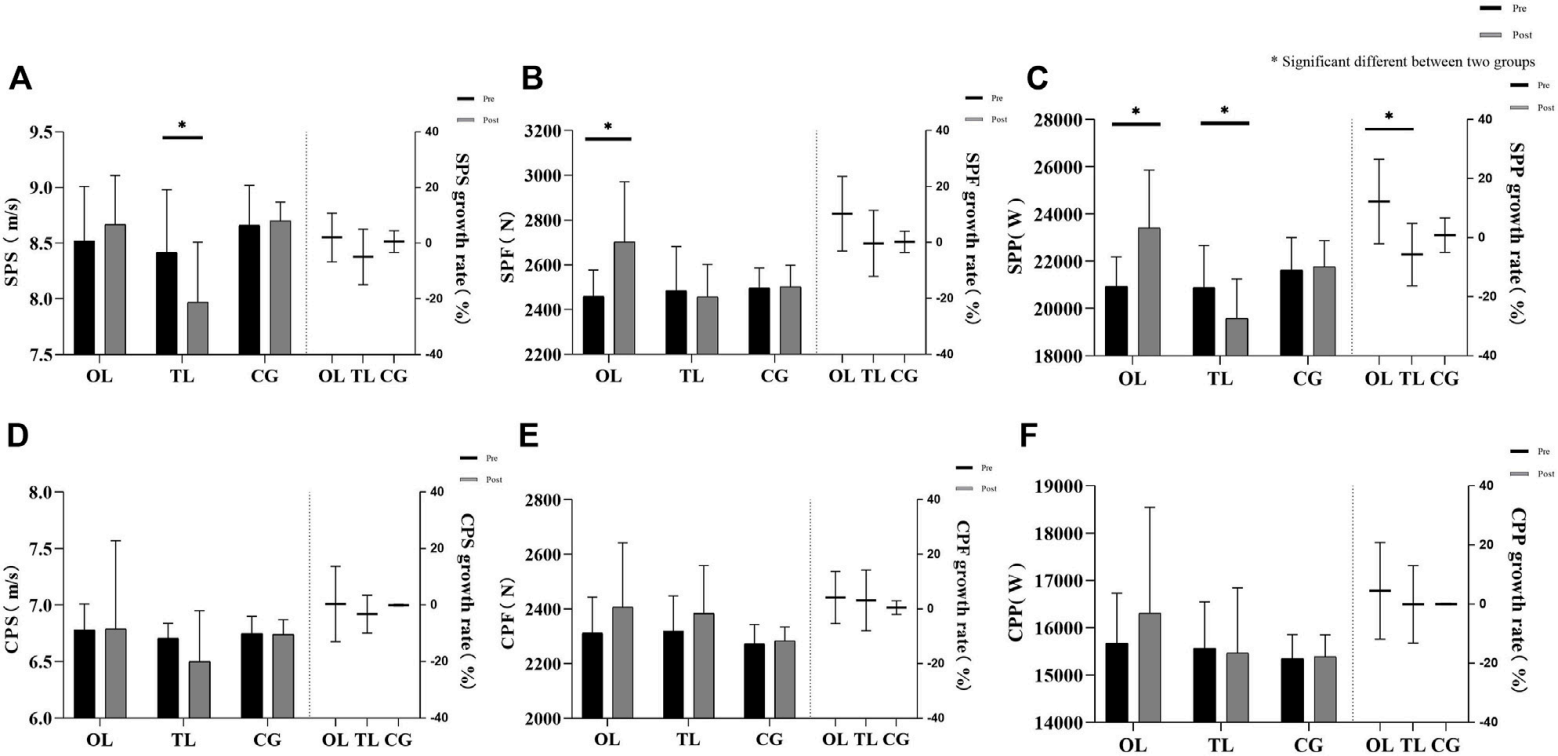
Results of one-way repeated-measures analysis of variance for punching ability: **(A)** single punching speed (SPS), **(B)** single punching force (SPF), **(C)** single punching power (SPP), **(D)** average speed of continuous punching (CPS), **(E)** average force of continuous punching (CPF), **(F)** average power of continuous punching (CPP).

**FIGURE 2:**
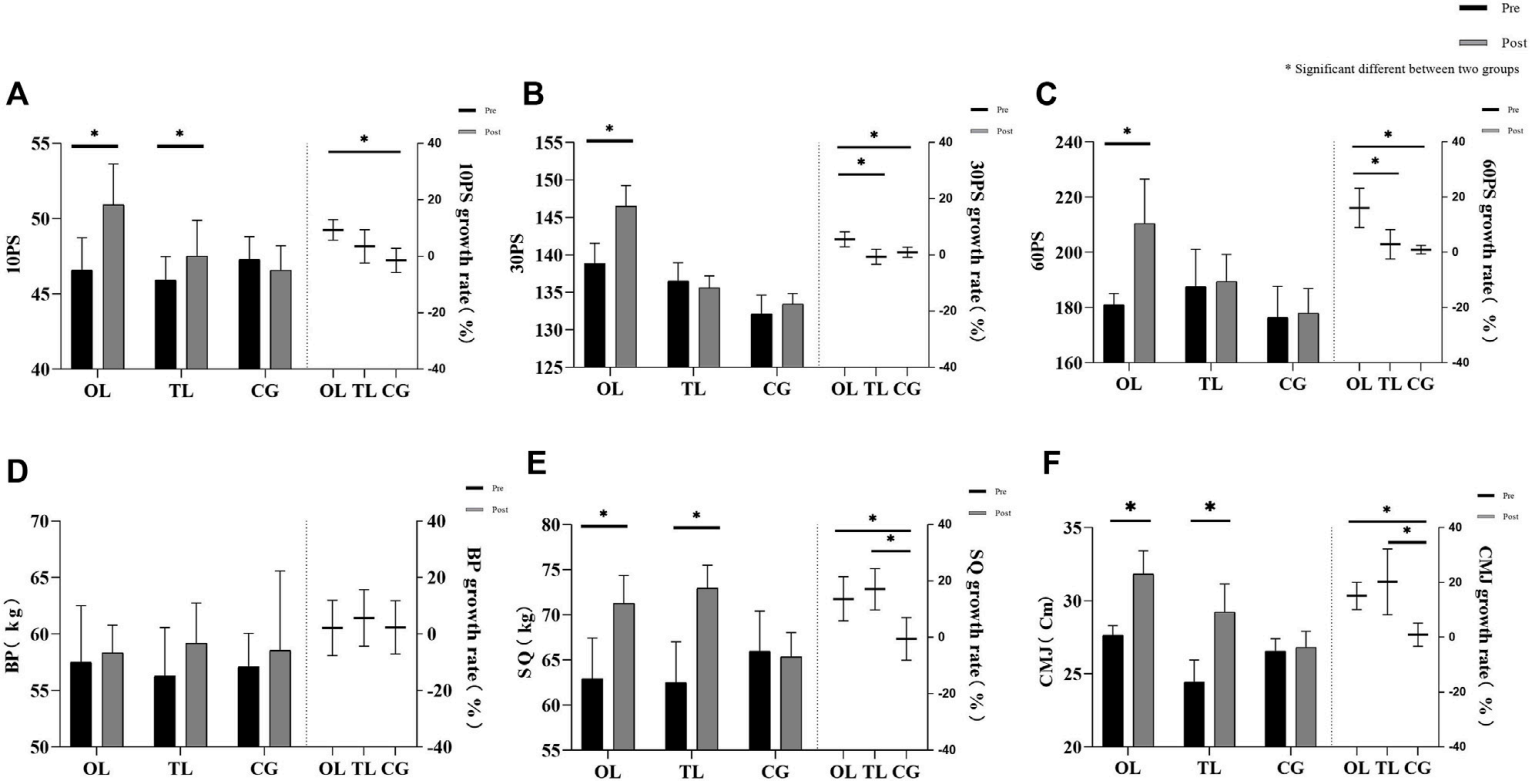
Results of one-way repeated-measures analysis of variance for sandbags punch and strength test: **(A)** number of sandbags punch in 10 s (10 PS), **(B)** number of sandbags punch in 30 s (30 PS), **(C)** number of sandbags punch in 60 s (60 PS), **(D)** 1 RM bench press (BP), **(E)** 1 RM squat (SQ), **(F)** counter movement jump (CMJ).

The effects of the six-week intervention on punching ability and strength in the three groups of subjects are shown in Figures 1, 2.

### 3.1 Within-group comparisons

OL: SPF increased (pre vs. post: 2458.96 ± 118.25 vs. 2703.33 ± 267.84; *p* = 0.003; 95% CI: −397.48 to −91.26); SPP increased (pre vs. post: 20936.76 ± 1247.06 vs. 23415.56 ± 2449.65; *p* = 0.001; 95% CI: −58.28 to −17.99); 10 PS increased (pre vs. post: 46.58 ± 2.15 vs. 50.92 ± 2.71; *p* = 0.000; 95% CI: −5.61 to −3.06); 30 PS increased (pre vs. post: 138.83 ± 2.72 vs. 146.50 ± 2.75; *p* = 0.000; 95% CI: −9.60 to −5.74); 60 PS increased (pre vs. post: 181.00 ± 4.05 vs. 210.25 ± 15.98; *p* = 0.000; 95% CI: −34.94 to −23.57); SQ increased (pre vs. post: 62.92 ± 4.50 vs. 71.25 ± 3.11; *p* = 0.000; 95% CI: −10.91 to −5.76); CMJ increased (pre vs. post: 27.65 ± 0.64 vs. 31.83 ± 1.59; *p* = 0.000; 95% CI: −5.31 to −3.03).

TL: SPS decreased (pre vs. post: 8.42 ± 0.56 vs. 7.97 ± 0.54; *p* = 0.026; 95% CI: 0.06–0.85); SPP decreased (pre vs. post: 20898.01 ± 1774.184 vs. 19582.39 ± 1668.526; *p* = 0.049; 95% CI: 0.094–40.39); 10 PS increased (pre vs. post: 45.92 ± 1.56 vs. 47.5 ± 2.39; *p* = 0.016; 95% CI: −2.86 to −0.31); SQ increased (pre vs. post: 62.50 ± 4.52 vs. 72.92 ± 2.57; *p* = 0.000; 95% CI: −12.99 to −7.85); and CMJ increased (pre vs. post: 24.42 ± 1.52 vs. 29.23 ± 1.91; *p* = 0.000; 95% CI: −5.81 to −3.53).

### 3.2 Between-group comparisons

The results of one-way repeated-measures analysis of variance showed that the improvement effect of SPP was significantly different across groups (*F* = 7.38; *p* = 0.004; *η* ^2^ = 0.402), and OL was significantly higher than that of TL (*p* = 0.012; 95% CI: 3.992–32.017). The improvement effect of 10 PS was significantly different in groups (*F* = 15.394; *p* = 0.000; *η* ^2^ = 0.583), and OL was significantly higher than CG (*p* = 0.000; 95% CI: 7.066–14.431). The improvement effect of 30 PS was significantly different in groups (*F* = 25.941; *p* = 0.000; *η* ^2^ = 0.702), and OL was significantly higher than TL (*p* = 0.000; 95% CI: 3.552–8.820) and CG (*p* = 0.001; 95% CI: 2.135–7.005). The improvement of 60 PS was significantly different between groups (*F* = 35.454; *p* = 0.000; *η* ^2^ = 0.763), and OL was significantly higher than TL (*p* = 0.001; 95% CI: 6.225–20.043) and CG (*p* = 0.000; 95% CI: 10.015–20.224). There were significant differences in improvement of SQ by group (*F* = 20.022; *p* = 0.000; *η* ^2^ = 0.645), OL (*p* = 0.002; 95% CI: 5.859–22.508) and TL (*p* = 0.001; 95% CI: 8.526–26.694) were significantly higher than CG. The improvement of CMJ was significantly different by group (*F* = 18.991; *p* = 0.000; *η* ^2^ = 0.633), OL (*p* = 0.000; 95% CI: 9.060–19.255) and TL (*p* = 0.002; 95% CI: 8.004–30.587) were significantly higher than CG.

## 4 Discussion

The objective of this study was to examine the impact of two distinct approaches to strength training, Optimal load training and traditional load training, on the punching ability and strength of boxers. Our findings suggest that optimal load training may better align with the specific demands of boxing and could be more effective in enhancing punching ability compared to traditional load training and routine team training. In contrast, traditional load training tends to focus more on improving strength.

### 4.1 The effect of optimal load training on punching ability

The OL made the subject’s power development more in line with the demands of boxing, enhance the mechanical work performed during punches, and indirectly improve the economy of the subject’s punches. “Economy” represents a complex interplay of physiological and biomechanical factors (Barnes and Kilding, 2015), “punching economy” is defined as the energy demand under the assumption that the athlete utilizes as many effective punches as possible in a boxing match. This led to the development of regeneration of the subject’s muscular endurance or explosive power. In contrast, the TL resulted in a decline in SPS, SPF, SPP, CPS, CPP, and 30 PS, and there was a significant decrease in SPS and SPP. The rationale behind this phenomenon was investigated in the context of the movement pattern and force generation characteristics of boxing. The primary objective of strength training for boxers is neuromuscular capacity training, rather than muscle hypertrophy training. For boxers, such as those engaged in bench press training, the objective is to link the chest, shoulders, and arms together to form a synergistic force, rather than solely to improve muscular strength. The traditional training load arrangement tends to focus primarily on improving maximum strength reserve, which may result in less emphasis on achieving the optimal ratio of force and speed. This approach can make it challenging to fully maximise punching ability.

The present study suggests that the ability of OL to enhance the subjects’ punching speed may be attributed to the effectiveness of the targeted training loads in this loading arrangement in improving the subjects’ neuromuscular coordination and facilitating greater fast muscle fibre recruitment (Pareja-Blanco et al., 2017a; Sañudo et al., 2020). The kinetic chain in boxing necessitates a force transfer from the lower limb to the arm and then from the core to the arm to complete the force release, due to the existence of this chain. Consequently, the quantity of power generated during the lower extremity stomp will have a direct impact on the rate of growth, and thus on the speed of the punch when the upper extremity performs the terminal release. The optimal load that produces the maximum power output can increase the force of the lower limb stirrups by improving the neural control of the muscles (Enoka, 2012; Heckman and Enoka, 2012), which is conducive to the increase of the punching speed. Furthermore, the speed of power transmission in the kinetic chain is also influenced by the excitation and inhibition of the motor nerve centre in the cerebral cortex and the coordination between the upper and lower limbs. Existing studies have demonstrated that training at the optimal load that can produce the maximum power output optimises the effect of these factors (González-Badillo et al., 2011).

Concurrently, OL augmented the subjects’ punching force, which may be attributed to the optimised ratio of load intensity and load volume in this loading arrangement, thereby aligning the training more closely with the demands of boxing. Furthermore, the training aimed at maximising the power output may effectively enhance the sensitivity of calcium ions in the myocytes, potentially leading to an enhancement of the muscle’s ability to contract rapidly. Concurrently, the muscle contraction force is augmented by modifying the pennation angle (Blazevich et al., 1985). The rationale behind training with optimal load is that it benefits the organism by recruiting more fast muscle fibres. This is because an appropriate load effectively prevents fatigue from occurring prematurely, and during training, subjects can maintain high neural excitability, which accelerates the conduction rate of action potentials in the nerves. This, in turn, provides the organism with the possibility of recruiting more fast motor units (Pareja-Blanco et al., 2017b), which is ultimately manifested in the enhancement of the subject’s punching force. Previous studies have also corroborated this hypothesis. A study on the efficacy of velocity loss based strength training, conducted by Galiano et al., demonstrated that the modulation of the amount of load based on the subject’s real-time state during strength training was an effective method for improving the subject’s maximal strength and lower extremity explosive power (Galiano et al., 2022). Folland and Sant Anielo et al. demonstrated in their study that suitable resistance training was beneficial for promoting changes in the pennation angle of muscle fibres to increase muscle contraction force (Folland and Williams, 2007; Santanielo et al., 2020).

As there is a significant correlation between punching power and punching force and speed, a change in either force or speed will cause a change in power (McGill et al., 2010; Loturco et al., 2014). In this study the SPF of the OL showed a significant increase and the results of the SPP were in line with this, also showing a significant increase.

The number of sandbag punches in 10, 30 s and 1 min can be used to assess the subject’s muscular endurance. Boxers must compete in multiple rounds, and the short and multiple power generation pattern and short intervals between rounds require that the boxers not only have the force and speed to perform well in the initial rounds of the competition, but also have the muscular endurance to cope with later rounds. Conversely, a reduction in punching speed and force will ultimately affect the performance of the match. However, according to related research, the ATP-CP system (Adenosine Triphosphate-Creatine Phosphate System) and CP reserve in human skeletal muscle can only meet approximately 50 intense muscle contractions. The ATP-CP system provides immediate energy for short bursts of high-intensity activity (up to 10 s) by using stored ATP and creatine phosphate in muscles, and the glycolysis is the breakdown of glucose into pyruvate in the cytoplasm, producing a net gain of 2 ATP. Under anaerobic conditions, pyruvate is converted into lactic acid, leading to muscle fatigue (Gottlieb et al., 2021). The number of punches a boxer throws in a match is much higher than 50, which requires a boxer to have a good anaerobic energy supply capacity and reserves of energy substances such as ATP and CP. In addition to the aforementioned attacking and defending movements, boxers must also perform a multitude of variable pace adjustment movements, which are dependent on the aerobic oxidative energy supply. Concurrently, boxers must be able to make rapid attacks at the appropriate time, which necessitates the ability to perform a large number of explosive force movements in short intervals. This inevitably results in the production of a considerable amount of lactic acid. The aerobic capacity is directly related to the efficiency of lactic acid removal, which directly affects the athlete’s physical status at the late stage of the match. Therefore, it is possible that the movement pattern and force generation characteristics of OL are more in line with the needs of boxing. This speculation suggests that the strength gains achieved through the training intervention may transfer effectively to actual boxing performance, potentially improving the economy of punching and indirectly optimizing the distribution of physical energy during the punching process. The present study demonstrated that the 60 PS of OL was improved by 16.08% ± 7.09%, which was the most significant indicator of improvement following the training intervention. This finding corroborates the aforementioned arguments.

### 4.2 The effect of optimal load training on strength

In terms of strength, the BP, SQ and CMJ tests reflected the subjects’ upper limb maximal strength, lower limb maximal strength and lower limb explosive power, respectively. Following the six-week training intervention, the strength indicators of OL and TL showed different degrees of improvement. Nevertheless, the improvement effect of TL was significantly superior to that of OL, and the pre- and post-test comparisons of the results of the SQ and CMJ demonstrated highly significant differences. In contrast, no significant difference was observed in the results of the BP of the OL.

This result demonstrates the distinction between the two contrasting approaches to load arrangement for strength training, in terms of the training effect and the adaptive changes produced on the organism. Strength training, as a fundamental component of the training regimen for boxers, must be aligned with the objectives of the boxing programme. On the one hand, it should facilitate the development of strength in all regions of the athlete’s body. On the other hand, it should also consider the need to enhance the athlete’s punching ability while maintaining the quality of their technical movements. It is essential to ensure that the load arrangement during training meets the needs of actual combat, not only to improve the strength reserve, but also to consider whether the strength acquired through training can improve punching ability without affecting the quality of the technical movements of the boxer. The findings of this study indicate that TL is primarily concerned with enhancing the subjects’ fundamental strength. During training, the strength of a specific muscle group in the subjects is augmented, yet the study fails to address the question of how to transform the subjects’ accumulation of basic strength into the output capacity required during competition. In boxing, both force and speed are considered to be winning factors. However, the application of pure strength training may result in an increase in the athlete’s body weight, impairment of muscle elasticity and a reduction in the rapid contraction of muscles, which in turn may lead to a decrease in the boxer’s punching speed (Behm et al., 2017). The optimal ratio of load intensity and volume in training is essential for effective punching. This is in line with the core idea of the optimal load training theory, which states that training under the optimal load is necessary to obtain the maximum power output.

Consequently, the training objectives of OL and TL diverge. TL may be more pertinent to the strength accumulation phase of a boxer’s sporting career, which can optimise their strength reserves and circumvent deficits. For elite boxers who have already established their own fighting style, OL will be more targeted and efficacious for them. In the context of the current boxing world, which is focused on intense confrontation, it is important to consider how to maximise the simultaneous increase in force and speed, improve the quality and quantity of effective punches, and delay fatigue. This is in order to ensure that the athletes are able to maintain their performance in the high-lactic acid state of sustained athleticism.

## 5 Limitation

In this study, the subjects were all female and further research is needed to determine whether the conclusions drawn are equally applicable to male boxers; due to conditions, this study was not able to modulate the female physiological cycle within the 6-week intervention, and athletes’ athletic performance may be influenced by hormone levels; this study only investigated the effects of OL and TL on boxers’ punching ability from an athletic performance perspective and did not include a mechanism-based study, which can be further demonstrated from a physiological mechanism perspective in future studies.

## 6 Conclusion

This one study suggests that TL may enhance the development of muscular strength, while OL appears more effective in improving punching ability, potentially facilitated by the individualized and targeted loading scheme. In terms of practical application, OL may be more applicable to elite athletes, as their strength has reached a point of stability at this stage, and they have developed their own athletic style, which requires a training arrangement that is more conducive to their needs. TL may be more applicable to the initial stage of an athlete’s career, which is conducive to the accumulation of strength.

## Data Availability

The raw data supporting the conclusions of this article will be made available by the authors, without undue reservation.
